# The Onset of Widespread Musculoskeletal Pain Is Associated with a Decrease in Healthy Ageing in Older People: A Population-Based Prospective Study

**DOI:** 10.1371/journal.pone.0059858

**Published:** 2013-03-29

**Authors:** Ross Wilkie, Abdelouahid Tajar, John McBeth

**Affiliations:** 1 Arthritis Research UK Primary Care Centre, Primary Care Sciences, Keele University, Keele, Staffordshire, United Kingdom; 2 Centre for Statistics in Medicine, University of Oxford, Oxford, United Kingdom; Universidad Pablo de Olavide, Centro Andaluz de Biología del Desarrollo-CSIC, Spain

## Abstract

**Objective:**

Chronic musculoskeletal pain is common in older adults but the nature of its relationship with ageing is unclear. The objective for this study was to test the hypothesis that the onset of widespread pain would be associated with a decrease in healthy ageing.

**Methods:**

Population-based prospective cohort study. A “healthy ageing” index was constructed across biomedical, physical, psychosocial and lay components. Analysis was performed with 2949 adults aged 50 years and over who had complete index scores at baseline, 3 and 6-year follow-ups.

**Results:**

At three and six year follow-up, 365 (16.8%) and 259 (14.3%) experienced the onset of widespread pain. The onset of widespread pain during the six-year period was associated with a 25% and a 46% decrease in healthy ageing index scores; this decrease was independent of age, sex, education, social networks, smoking status, alcohol consumption and physical inactivity. The decrease in healthy ageing attenuated to 20% and 39% following adjustment for diagnosed musculoskeletal conditions and analgesic and non-steroidal use.

**Conclusions:**

The onset of widespread pain was associated with a decrease in healthy ageing throughout the six-year period. When pain increased over time, the markers of unhealthy ageing increased also. Strong analgesia was associated with unhealthy ageing. Research could now usefully test whether early identification, improved treatment and prevention of pain prior to old age may facilitate healthy ageing.

## Introduction

Chronic musculoskeletal pain is one of the most common disorders in older people [1]. Whilst chronic pain in older people is often attributed to osteoarthritis and allied disorders, it is clear that this is also a problem of chronic pain *per se* [2] which is not necessarily associated with advanced radiographic degeneration in the joint in which the symptoms occur [3] and commonly occurs in multiple bodily sites including non-joint sites [4]. The World Health Organization highlights pain as a key driver of the impact of musculoskeletal conditions to the global burden of disability [5]. Persons with pain that is widespread throughout the body have worse outcomes when compared to those with less diffuse pain and those who are pain free. In older people widespread pain is common [1] and is strongly associated with poor outcomes across multiple health domains including cognitive functioning, sexual functioning, and falls [6]–[8]. With population ageing a major concern is that increased longevity is not accompanied by active and healthy ageing, which enables individuals to maintain independent lives until old age [9]. Healthy ageing can be characterized by functional independence, a multi-faceted state, involving preservation of biomedical, physical and psychosocial health that enables cognitive, physical and mental wellbeing, social participation and improved quality of life [10], [11]. Identifying factors that negatively impact on healthy ageing but that are amenable to change, is important. While conceptualizing and operationalizing the complexity of multiple outcomes is challenging, the construct of healthy ageing provides a useful method to capture outcomes across multiple domains [10].

This is the first longitudinal study to examine the association between musculoskeletal pain and healthy ageing. The study tested the hypothesis that the onset of widespread musculoskeletal pain would be associated with a decrease in healthy ageing.

## Methods

### Study Design

This study draws on a well-established population-based cohort study (the North Staffordshire Osteoarthritis Project, NorStOP) of the long-term prognosis of musculoskeletal pain in older people [12]. The sampling frame was individuals aged ≥50 years registered with six general practices in North Staffordshire, United Kingdom where 98% of the population are registered with a general practice. These registers provide convenient sampling frames of the local general population and allow survey data to be linked to medical record data. The North Staffordshire Local Research Ethics Committee granted approval and all participants gave written consent to participate.

### Participant Flow

**Figure 1:**
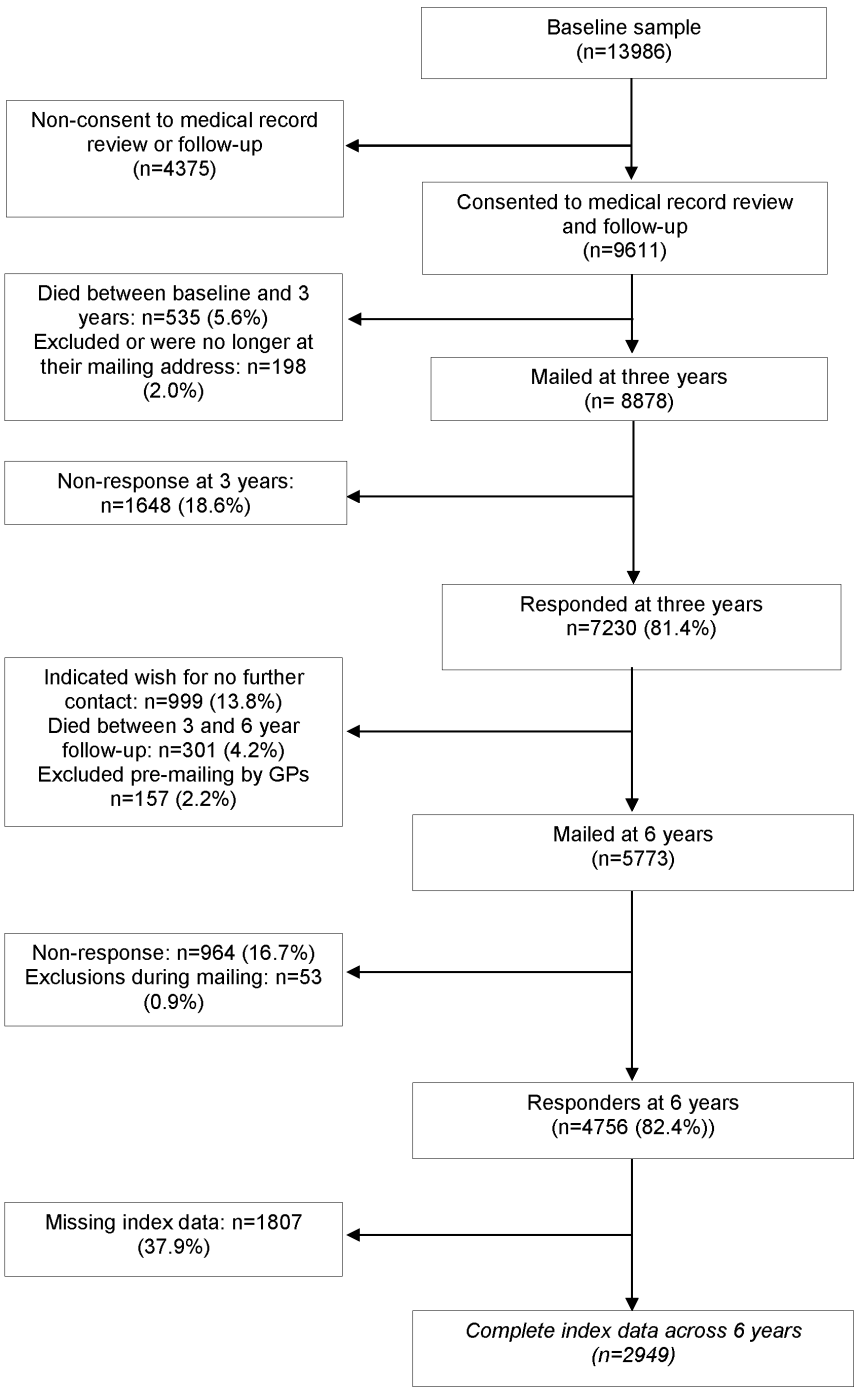
Flow diagram of participants.

A total of 13986 individuals returned a questionnaire of whom 9611 (68.7%) provided consent for the study team to access their medical records. Participants were followed up 3 and 6 years after the date of their baseline questionnaire. A total of 836 (8.7%) participants died over the six year period leaving 8775 participants. Of those 2949 (20.1%) provided complete data at baseline, 3 and 6 year follow-ups (n = 2949) (Figure 1). This represents 30.1% of those who provided consent for medical record review and were alive at 6 year follow up and could have returned a questionnaire (see below for data on number of deaths). This response rate is comparable with other population based cohorts (e.g. Asset and Health Dynamic Survey Among the Oldest Old (AHEAD) [13]). Compared to those subjects who had moved address, withdrew from the study or had incomplete data (n = 5826), those included in the analysis were younger (mean age: 64.3 cf cf 61.7; p<0.001), more likely to have gone onto further education (9.8% cf 11.7%; p<0.001), have better mental and physical health-related quality of life scores (Mean SF-12 [14] mental component: 55.1 cf 52.2 (p<0.001) and physical component (Mean SF-12 [14] physical health scores: 47.7 cf 42.0; p<0.001)), have higher baseline index scores indicating more healthy ageing (74.6 cf 78.5; p<0.001) and lower levels of cognitive impairment (49.7% cf 39.1%; p<0.001) and depression (possible/probable cases of depression: 24.0% cf 14.3%; p<0.001) (Table 1). However there was no difference for gender (male: 45.3% cf 44.8%; p = 0.72) or in the relationship between widespread pain and healthy ageing index score at baseline (i.e. proportional difference (i.e. the proportion (%) that the healthy ageing index score is higher in those with widespread pain compared to those with no pain): 126% (116%, 138%) in the attrition group cf 127% (114%, 141%) in the analysed sample). Those who died after baseline (n = 836) had significantly lower baseline index scores (57.7; p<0.001); were older (mean age 73.9 years), had lower levels of health (Mean SF-12 mental health component: 47.3; Mean SF-12 physical health component: 30.9), higher levels of cognitive impairment (61.8%) and depression (34.8%) and a weaker relationship between widespread pain and health ageing (proportional difference: 87% (68%, 108%)) compared to those included in the analysis.

**Table 1:** Characteristics of those included in the analysis, those who withdrew or had incomplete data and those who died during the 6 year study period.

	Overall(n = 2949)	Withdrawn or incomplete data(n = 5826)	Died(n = 836)	P value
Age (years)*	61.7 (0.25)	64.3 (0.51)	73.9 (0.25)	<0.001
Healthy Ageing Index score*	78.97 (0.38)	70.3 (0.39)	55.9 (0.38)	<0.001
**Gender (Male)**	1334 (45.3%)	2612 (44.8%)	486 (58.1%)	<0.001
**Education (Further education)**	478 (16.4%)	661 (11.7%)	67 (8.3%)	<0.001
Physical health*	47.7 (0.38)	42.0 (0.38)	30.9 (0.57)	<0.001
Mental health*	55.1 (0.20)	55.2 (0.88)	47.3 (0.29)	<0.001
**Cognitive impairment**	1154 (39.1%)	2894 (49.7%)	517 (61.8%)	<0.001
**Depression**	422 (14.3%)	1398 (24.0%)	293 (34.8%)	<0.001

All values are n (%) except * which are median (standard error).

Kruskal Wallis test for age and index, chi square for gender, education and social network.

### Pain Assessment and Classification

Participants who experienced pain lasting for one day or longer in the past month were asked to indicate the site of their pain by shading on a blank body manikin (front and back views). Based on their pain reports participants were categorized into one of three groups: “no pain”, “regional pain” or “widespread pain”. Widespread pain was classified according to the American College of Rheumatology (ACR) criteria used in their definition for fibromyalgia [15] which require pain to be present in the left and right hand sides of the body, above and below the waist, and in the axial skeleton. The regional pain group was those subjects reporting pain that did not satisfy the criteria for widespread pain, and the no pain group were those who were pain free. These methods to determine the location and extent of pain are commonly used in population based studies of pain, and have been shown to be valid and reliable [16].

Those participants who reported no pain or regional pain at baseline or three years and who reported widespread pain at three and six year follow up respectively, were classified as having “new onset of widespread pain”.

### Healthy Ageing Index

#### Index development

Healthy ageing has been defined in a number ways: biomedical models emphasize the absence of disease and maintenance of physical and mental functioning; psychosocial models focus on life satisfaction and social participation; and lay models highlight accomplishment and contribution to life. Models that combine all three approaches better predict poor outcomes than one-dimensional approaches and have been proposed to evaluate independence in older adults [17]. The key aspects of the different approaches are health, psychological factors, social roles and activities, finances, social relationships and neighborhood factors [10]. Healthy ageing can be measured as an index of the maintenance and continued achievement of these different aspects and an index can be constructed using methods successfully employed in biomedical models of frailty that capture the rate of deficit accumulation [18], [19].

NorStOP was designed to measure the impact of pain in older adults and capture multiple constructs of ageing [12]. Validated instruments (e.g. Hospital Anxiety and Depression scale [20]) were used to measure some constructs (e.g. depression, anxiety) and psychometric testing of the self-report questionnaire indicated that the data on impairments and function were accurate [12], [21]. A total of 33 variables that showed a strong age-related decline were included in the healthy ageing index (Table 2). These covered biomedical, psychological, physical and social systems and covered the different aspects of healthy and successful ageing proposed by Bowling and Dieppe [10]. The score range of individual variables was from 0 to 1. The total score across all variables was a simple count of all variables and ranged from 0 to 33 [18], [19]. Participants total score was expressed as a healthy ageing index by dividing the total score by the maximum score (i.e. 33) and expressed on a scale ranging from 0 to 100. Higher index scores indicated healthy ageing and were calculated for each participant at baseline, 3 and 6 years.

**Table 2:** Healthy ageing index constituent variables.

Domain of healthy ageing	Variables	Score (0–1)
**Physical function**	Limitation in vigorous activitiesLimitation in moderate activitiesLimitation in lifting or carrying groceriesLimitation climbing one flight of stairsLimitation bending, kneeling or stoopingLimitation walking half a mileLimitation bathing and dressing	For each item:1– No limitation0.5– Limited a little0– Limited a lot
**Biomedical**	Self-rating of health	1– Excellent, 0.75– Very good, 0.5– Good, 0.25– Fair, 0 - Poor
	Unhealthy weight	1 = Normal weight (20–24.9)0.5 = Overweight (25–29.9)0 = Underweight (<20) & Obese (30+)
	Chest problemsHeart problemsDiabetesDeafnessProblems with eyesightRaised blood pressureSuffered a fallDizziness or unsteadinessWeakness in an arm or leg	For each item:1- Absent0-Present
	Cognitive impairment	1– Not impaired0- Impaired
**Psychological**	Anxiety	1– Non-case, 0.5– Possible, 0 - Probable
	Depression	1– Non-case, 0.5– Possible, 0– Probable
	Sleep problems	1– No sleep problems0 - Any sleep problem
**Lay**	Accomplishment of daily activities	1-No limitation in accomplishing daily tasks0-Not accomplishing daily tasks
	Feeling of calm and peaceFeeling of having a lot of energy	For each item:1– All the time0.8– Most of the time0.6– A good bit of the time0.4– Some of the time0.2– A little bit of the time0– None of the time
	Financial strain	1–Manage/comfortable0- Strain/have difficulty
**Perceived social** **participation**	Restrictions in mobility within homeRestrictions in mobility out with the homeRestrictions in self-careRestrictions looking after the homeRestrictions looking after belongingsRestrictions communicating with othersRestrictions in social activities	For each item:1– Not restricted0 - Restricted

At each time point the healthy ageing index score ranges from 0 to 100; higher scores indicate a greater level of “healthy ageing”. The formula to calculate the score at each time point is: (total score/33)*100.

#### Index validity

To test the ability of the index to measure healthy ageing, the relationship with mortality was examined. Lower index scores were associated with an increased risk of mortality. The median baseline healthy ageing index scores for the 836 participants who died during the six year period was significantly lower than for those who were included in the analysis (n = 2949) (57.7 cf 79.0; p<0.001).

### Putative Confounders

Demographic factors included in the analysis as potential confounders were age, sex, social networks (Berkman-Syme Social Network Index [22], score range 0–4, categorised as *high/medium* (score 3 to 4) or *low* (score 0 to 2) network) and educational attainment (completed high school only; went on to further education). Behavioural factors included were smoking status (never/previous/current), frequency of alcohol consumption (monthly or weekly/never or yearly/daily) and physical inactivity (two items: frequency of going to activities outside the home and frequency of going for a walk for at least ten to fifteen minutes (both categorised as daily, every other day, twice per week, Less than twice per week, not at all)). To assess the impact of clinical factors (diagnosed musculoskeletal disorders and medication use) the primary care medical records of participants were interrogated. Diagnoses of chronic musculoskeletal conditions (osteoarthritis and inflammatory arthropathies) were recorded using Read codes [23]; these are used in primary care by practitioners to record morbidity data on clinical computer systems. The Read codes cross-map to ICD9/ICD-10 (for diseases), OPCS-4 (for operations, procedures and interventions), BNF and ATC (for drugs). Read code N04 was used to identify the diagnoses of rheumatoid arthritis or any other inflammatory arthropathy and N05 for osteoarthritis [23]. Pain analgesia was categorized using a validated model based on the strongest prescribed analgesia during the six year period (i.e. none, basic (e.g. paracetamol), weak, moderate, strong, very strong (e.g. morphine) [24]). The prescription of non-steroidal anti-inflammatory drugs was recorded as a binary variable (prescribed/not prescribed). This consultation data has been shown to provide accurate measurements of morbidities, and prescribed medications [25].

### Statistical Analysis

The baseline characteristics were described overall and stratified by baseline pain status. Differences between the baseline pain groups for healthy ageing index score and age were tested using Kruskal Wallis test and for education, social network, smoking, alcohol consumption, physical inactivity, diagnosis of musculoskeletal condition, prescription of analgesia and anti-inflammatories using a chi-square test. The distribution of the healthy ageing index score had moderate skewness and kurtosis (baseline index: skewness 1.09; kurtosis 4.01) and was log transformed. The results were presented as percentage change in healthy ageing index score. This was calculated from the beta coefficients (β) of each variable in the model using the formula (100* (exp(β) -1)). To test the study hypothesis that the onset of widespread pain would be associated with a decrease in healthy ageing index score, a mixed modelling regression approach was used to analyse the longitudinal data of this study [26]; data at three years was included to examine if change in healthy ageing index scores was linear. This strategy accounts for within participant correlation and between participant variations in healthy ageing index scores and takes into account the correlation between measurements of the same participant. First, the mean percentage change in healthy ageing index score associated with *time* was estimated. Then pain status was entered into the model as a time-varying variable (i.e. over the follow up period participants can move between pain states). The mean percentage change in healthy ageing index score associated with the onset of widespread pain was then estimated using published methods [26]. For example, the mean percentage change in healthy ageing index score among participants with no pain at baseline who reported widespread pain at follow up = *time*+(mean percentage change for widespread pain – mean percentage change for no pain). These mean percentage changes were then adjusted for potential confounders: socio-demographic, behavioural factors, use of pain analgesia and non-steriodals, and diagnoses of chronic musculoskeletal conditions. Model goodness of fit was assessed by Akaike Information Criteria (AIC) with lower values indicating improved model fit.

Sensitivity to subject attrition and missing data was examined via probability-weighted analysis for survey data [27]. As there were no differences in inference between the original and weighted analyses the former are reported.

All analyses were conducted using Intercooled Stata version 9.2 (StataCorp, College Station TX).

## Results

### Participant Characteristics

**Figure 2:**
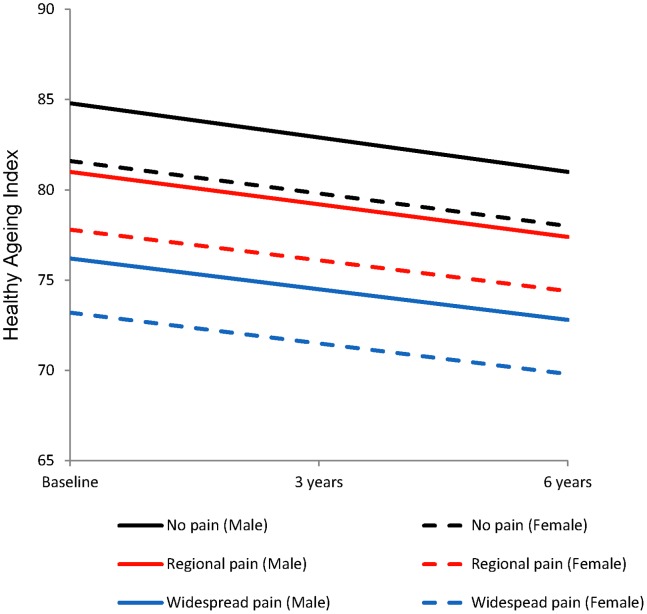
Mean healthy ageing index scores over 6 years for women and men by pain status, adjusted for age, education and social networks.

At baseline, median (Standard Error (SE)) age was 61.7 (0.25) years, 54.7% were women, 83.8% had a high school education only, and 48.1% had medium/low social networks (Table 3). A total of 873 (29.6%) participants had no pain, 780 (26.4%) had widespread pain and 1296 (43.9%) had regional pain (of this group, 442 (34.1%) had one to three areas of pain, 442 (34.1%) had four to six areas of pain and 412 (31.8%) had more than six areas of pain; 19 (1.5%) had spinal pain only, 361 (27.9%) had lower limb pain only, 181 (14.0) had upper limb pain only; 127 (9.8%) had spinal pain and upper limb pain, 238 (18.4%) had spinal and lower limb pain, 338 (26.1%) had upper and lower limb pain and 32 (2.4%) had unilateral upper and lower limb and spinal pain). Reporting regional or widespread pain did not increase with age (p = 0.17) (Table 3). Women were more likely to report widespread pain than men (29.9% cf 23.1%; p<0.001). The median healthy ageing index score was 79.0 (SE 0.33) at baseline which significantly decreased at 3 (77.3 (0.34)) and 6 (76.2 (0.36)) year follow-up (p<0.001). The median healthy ageing index score at baseline for those with no, regional and widespread pain was 87.4 (0.33), 78.9 (0.47) and 68.2 (0.78) respectively. For all pain categories, women had lower healthy ageing scores across the six years, than men (Figure 2).

**Table 3:** Subject characteristics at baseline overall and by baseline pain status.

	Overall(n = 2949)	No pain(n = 873)	Regional pain(n = 1296)	Widespread pain(n = 780)	P value
Age (years)*	61.72 (0.25)	61.94 (0.25)	61.91 (0.25)	61.30 (0.51)	0.17
Healthy Ageing Index score*	78.97 (0.33)	87.06 (0.33)	78.90 (0.47)	68.24 (0.78)	<0.001
**Gender**					
(Female)	1592 (54.71)	431 (51.68)	685 (52.85)	476 (61.03)	<0.001
**Education**					
(no further education)	2415 (83.77)	673 (81.67)	1072 (83.49)	670 (86.45)	0.033
**Social network**					
(med/low)	1,419 (48.1)	405 (58.61)	620 (56.83)	394 (58.81)	0.644
**Smoking**					
PreviousCurrent	1190 (40.9)369 (12.7)	317 (38.0)97 (11.6)	545 (42.1)161 (12.4)	328 (42.1)111 (14.2)	0.06
**Alcohol**					
Weekly/monthlyNever/yearlyDaily	1604 (55.2)618 (21.3)684 (23.5)	461 (55.0)179 (21.5)194 (23.7)	727 (56.3)242 (18.7)329 (25.1)	416 (53.1)197 (25.3)166 (21.3	0.01
**Physical inactivity: Frequency of getting out and about**					
All daysMost daysSome daysFew/no days	1135 (39.6)1007 (35.1)530 (18.5)198 (6.9)	383 (46.7)301 (36.7)105 (12.8)32 (3.9)	526 (41.2)435 (34.1)241 (18.9)75 (5.8)	226 (29.3)271 (35.1)184 (23.8)91 (11.8)	<0.001
**Physical inactivity: Walking for 5–10 minutes**					
Every dayEvery other/twiceweekly<1 weekNever	436 (15.2)1365 (47.6)589 (20.5)477 (16.6)	141 (17.2)447 (54.6)142 (17.3)89 (10.9)	199 (15.6)616 (48.2)255 (19.9)209 (16.3)	96 (12.5)302 (39.3)192 (25.0)179 (23.3)	<0.001
**Diagnosed musculoskeletal** **condition**	724 (24.6)	90 (10.3)	358 (27.6)	276 (35.4)	<0.001
**Level of prescribed analgesia during the six year period**					
NoneBasicWeakModerateStrongVery strong	1283 (43.5)301 (10.2)365 (12.4)251 (8.5)715 (24.3)34 (1.2)	533 (61.1)88 (10.1)101 (11.6)40 (4.6)110 (12.6)1 (0.11)	533 (41.1)155 (12.0)160 (12.4)117 (9.0)314 (24.2)17 (1.3)	217 (27.8)58 (7.4)104 (13.3)94 (12.1)291 (37.3)16 (2.1)	<0.001
**Prescribed non-steriodal** **anti-inflammatories during** **the six year period**	1197 (40.6)	237 (27.1)	562 (43.4)	398 (51.0)	<0.001

All values are n (%) except * which are median (standard error).

Kruskal Wallis test for age and index, chi square for gender, education and social network.

### The Association between the Onset of Widespread Pain and Healthy Ageing Index Score

**Figure 3:**
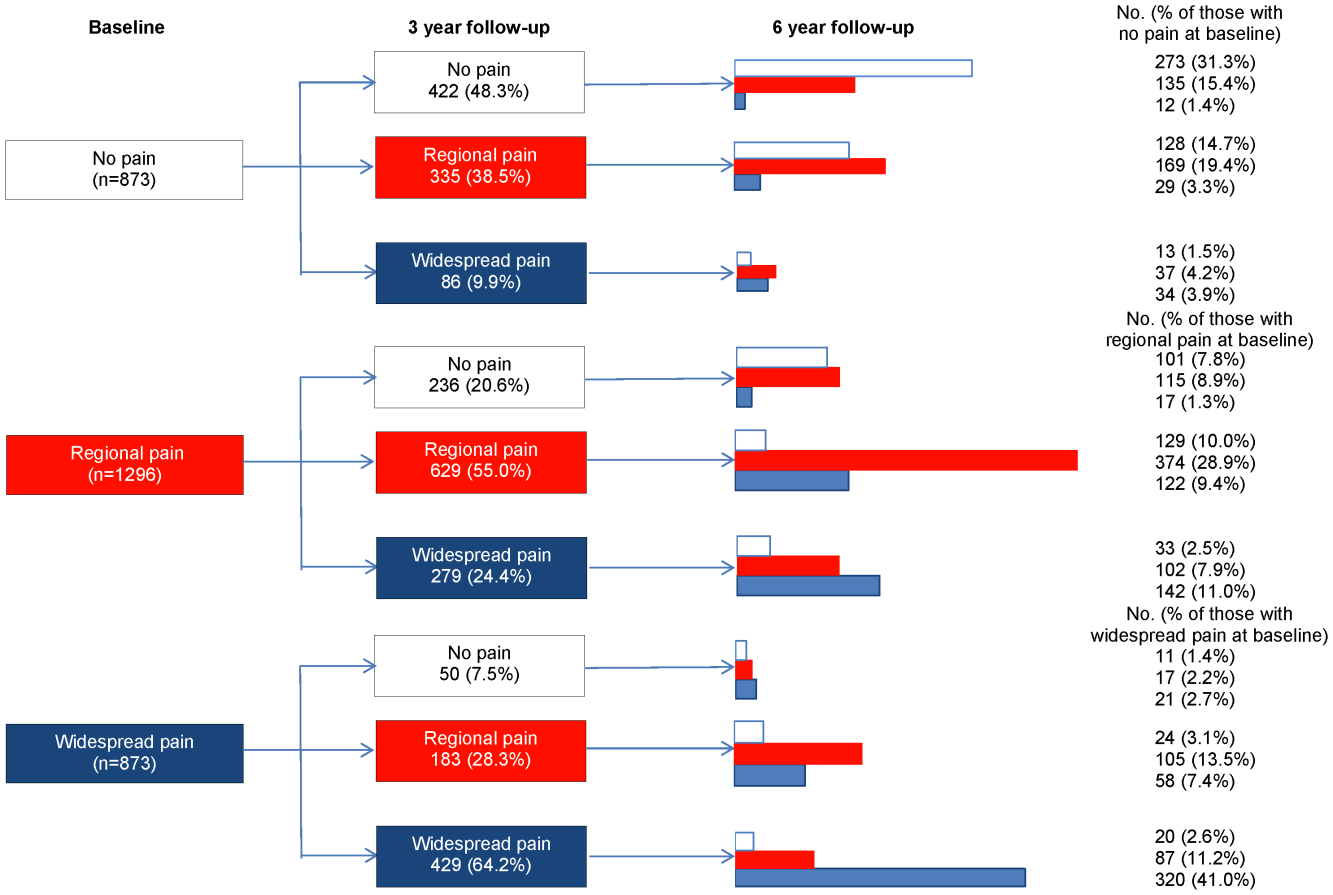
Changes in musculoskeletal pain classifications at 3 and 6 year follow-up.

Independent of pain status mean healthy ageing index scores decreased by 6.2% over the six year follow up period (exp(0.06)-1)*100; i.e. 0.06 was the beta coefficient decrease in the healthy ageing index score (log of healthy ageing index) which corresponded to a 6 year increase, the formula above transforms the beta coefficient to a percentage change in the original healthy ageing index score at baseline) (Table 4, Model I). At three and six year follow-up, 365 (16.8%) and 259 (14.3%) had experienced the onset of widespread pain (Figure 3). The onset of widespread pain was associated with a significant decrease in healthy ageing across the 6 years of follow up (p<0.05). Among subjects with no pain the onset of widespread pain was associated with a total decrease in healthy ageing index score of 46.7% (i.e. the decrease in healthy ageing index score for those with widespread pain compared to those with no pain was 40.5%+the decrease of 6.2% associated with time) (Table 4, Model I, which includes only time and pain). This association remained unchanged when adjusted for age, sex, education, social networks, smoking status, alcohol consumption and physical inactivity (Table 4, Model II). Further adjustment for analgesic use, non-steroidal use, and diagnosis of musculoskeletal conditions attenuated the association, with the onset of widespread pain associated with a 39.0% decrease in healthy ageing index score. In the final model, increasing age, female gender, low educational attainment (i.e. school education only), medium/low social networks, smoking, physical inactivity, prescribed analgesia, prescription of non-steroidal anti-inflammatories and the diagnosis of a musculoskeletal condition were also associated with a decrease in healthy ageing index score (Table 4, Model III).

**Table 4:** Longitudinal relationship between pain status and healthy ageing over six years.

	Model (I)	Model (II)	Model (III)
**Intercept**	97.17(0.013)(97.15, 97.20)***	97.98(0.046)(97.89, 97. 93)***	97.99(0.052)(97.91, 98.08) ***
**TIME**	−6.2%(0.004)(−5.1%, −7.2%)***	−6.2%(0.004)(−5.1%, −7.2%)***	−6.2%(0.005)(−5.0%, −7.3%)***
***Pain status***			
**No pain**	Referent	Referent	Referent
**Regional pain**	−19.7%(0.012)(−17.3%, −23.3%)***	−20.9%(0.013)(−18.5%, −24.6%)***	−19.1%(0.016)(−15.6%, −22.9%)***
**Widespread pain**	−40.5%(0.014)(−36.3%, −43.3%)***	−39.5%(0.016)(−34.9%, −43.9%)***	−32.8%(0.018)(−28.1%, −37.7%)***
**Gender (female)**	−	−9.1%(0.021)(−4.7%, −9.1%)***	−8.1%(0.023)(−3.9%, −12.5%)***
**Age (years)**	−	−1.4%(0.001)(−1.1%, −1.6%)***	−1.1%(0.001)(−0.8%, −1.3%)***
**Social network (med/low)**	−	−5.7%(0.021)(−1.5%, −10.2%)***	−5.5%(0.023)(−1.6%, −9.7%)***
**Education** **(School education only)**	−	−6.9%(0.028)(−1.2%, −10.1%)***	−3.5%(0.031)(−2.0%, −8.9%)***
**Smoking**	−	−8.9%(0.015)(−5.8%, −12.3%)***	−7.2%(0.013)(−4.2%, −10.2%)***
**No Alcohol**	−	4.1%(0.03)(−1.3%, 9.6%)	5.1%(0.03)(−0.03%, 10.5%)
**Daily alcohol**	−	−3.3%(0.03)(−8.6%, 1.6%)	−3.0%(0.02)(−8.3%, 1.6%)
**Getting out of the house**	−	−19.1%(0.012)(16.2%, 21.9%)***	−16.8%(0.009)(−14.2%, −19.5%)***
**Walking for 10**–**15 minutes**	−	−5.3%(0.008)(−3.7%, −7.3%)***	−3.9%(0.017)(−2.3, −5.7) ***
**Analgesic use**	−	−	−9.1%(0.008)(−7.8%, −10.6%)***
**Non-steriodal use**	−	−	−0.2%(0.001)(−0.1%, −0.4%)***
**Diagnosis of musculoskeletal conditions**	−	−	−112.1%(0.024)(−6.9%, −17.7%)***
**RANDOM EFFECT**			
Level 2 variance (Individuals)	0.28(0.009)(0.26,0.30)	0.21(0.007)(0.20,0.23)	0.18(0.007)(0.17, 0.20)
Level 1 variance (Repeated measurements)	0.103(0.002)(0.099,0.106)	0.10(0.002)(0.010,0.11)	0.10(0.002)(0.10, 0.10)
**Akaike information criterion (AIC)**	11229	8503	8171

All values are followed by standard error and 95% confidence intervals. Model I: Addition of painstatus as a time-varying variable (i.e. over the follow up period subjects can move between pain states). Model II: Adjustment for potential confounders: age, gender, educational attainment, social networks, smoking status, alcohol consumption and physical inactivity. Model III: Further adjustment for use of pain analgesia and non-steroidals, and diagnoses of chronic musculoskeletal conditions’.

*** p<0.001, CI Confidence interval, – Variable not included.

Among subjects with regional pain the onset of widespread pain was associated with a 24.8% decrease in healthy ageing index score (i.e. the decrease in healthy ageing index score for those with widespread pain compared to those with regional pain was 18.6% +6.2% (time)). The decrease in healthy ageing index score was attenuated to 19.9% following adjustment for diagnosed musculoskeletal conditions and analgesic and non-steroidal use.

For completion, we also report the associations with the onset of regional pain. At three and six year follow-up, 335 (38.5% of those with no pain at baseline) and 267 (38.0% of those with no pain at three year follow-up) had experienced the onset of regional pain (Figure 3). The onset of regional pain was associated with a significant decrease in healthy ageing across the 6 years of follow up (p<0.05). Among those subjects reporting no pain, the onset of regional pain was associated with a total decrease in healthy ageing index score of 25.9% (i.e. decrease in healthy ageing index score for those with regional pain compared to those with no pain was 19.7+ the decrease of 6.2% associated with time) (Table 4, Model 1). Adjustment for age, gender, education, social networks, smoking status, alcohol consumption, physical inactivity, diagnosed musculoskeletal conditions, analgesic and non-steriodal use had little effect (i.e. total decrease of 25.3%).

Between-subject variation explained 64.3% of the total healthy ageing index score variance (R^2^ for between subject variation/R^2^ for total variance; 0.18/0.28) while within subject variation explained 35.8%. The addition of diagnosed chronic musculoskeletal conditions and use of analgesics and non-steroidals improved model goodness of fit by 4.0% (i.e. AIC decreased from 8503 to 8171) (Table 4, Model III).

## Discussion

### Summary of Findings

This study has shown that among older people, the onset of widespread pain was associated with a composite measure of unhealthy ageing. For all three pain states, older adults aged less healthily across the six-years of follow-up. However the onset of widespread pain during the 6 year period, independent of age and time, was associated with a significant decrease in healthy ageing index scores. Socio-demographic factors were significantly associated with a decrease in healthy ageing, particularly female gender. In this study adjustment for analgesia attenuated the relationship between widespread pain and healthy ageing index score. However, the extent to which analgesia was a marker of severe pain or was ineffective in relation to the impact of pain must await long-term intervention studies.

There are no studies with which to directly compare these data. It is clear that pain impacts on a number of body systems and, particularly widespread pain, is associated with poor outcomes across biomedical, psychological and social domains [28]–[29]. The novelty of this report is the application of a healthy ageing index as a broader outcome which captures the diverse and complex impact of pain across multiple domains. Other studies have reported significant associations between factors such as alcohol use [30], cognitive function [31], and early life influences such as educational attainments [30] and healthy ageing. The mechanisms underpinning these associations are unclear. In relation to pain the association may be due to a number of biological and psychological responses to pain which impact on other body systems. For example, persistent exposure to pain down-regulates critical components of the hypothalamic pituitary adrenal stress axis. Patients with fibromyalgia, a disorder characterized by chronic widespread body pain [15] have reduced levels of urinary and plasma cortisol, cortisol feedback resistance and hyporesponsive pituitary function [32], as well as abnormal immune response. As cortisol is responsible for downstream immune-suppression, hypocortisolism results in atypical elevations in cytokines IL-8, TNF-α, IL-1 and IL-6 [33] which may in turn be associated with unhealthy ageing [34].

### Strengths and Limitations of the Study

This study has a number of strengths. The sample was derived from a large population-based study of older community dwelling individuals. Data was collected prospectively and allowed the relationship between the onset of widespread pain and changes in healthy ageing index scores to be determined. The concept of ’healthy ageing’ captures multiple outcomes and the complexity and quality of increasing longevity [10]. The concept of healthy ageing is evolving and has similarities with other multidimensional constructs in ageing research, which have attained consensus, been adopted and advanced the field (e.g. successful ageing and frailty) [18], [19]. The approach taken in this study moves beyond the purely biomedical approach of frailty and incorporates the biomedical, psychosocial and lay approaches of the healthy ageing concept, which capture relevance to older adults and can be used to evaluate outcomes of health promotion in older populations [10]. Similar multi-dimensional models of healthy ageing, that have derived multidimensional indices [19] have strong links with quality of life and can be considered to capture the construct.

Study limitations were: The healthy ageing index was constructed using self-report data. Although this is susceptible to measurement error, the self-report of impairments and functional problems (e.g. falls) have been shown to be accurate in older adults [35] and validated instruments (e.g. Hospital Anxiety and Depression scale [20]) were used to measure many of the constructs. The method for identifying medication use is limited to those prescribed and does not include over the counter medication. This may misclassify responders to no pain medication when they are using over the counter medication and underestimate the association between medication use and the healthy ageing index score. It may also underestimate the extent of attenuation between pain and healthy ageing index score, when adjusted for medication use. This is the first study which has operationalized healthy ageing as an accumulation of deficits. The method of selecting all items based solely on their relationship with age may be a limitation. Selection of items using factor analysis and subsequent testing of internal consistency would provide further support that the five constructs of the index were being measured. Further testing of the reliability and responsiveness would provide further information on the index’s psychometric properties. The index has been examined for construct validity (i.e. healthy ageing index scores link with ageing and mortality) and requires further testing of internal and external validity (i.e. derivation of the healthy ageing index in another dataset using the same 33 variables with additional psychometric testing of the construct). Additional analyses of the underlying construct and of the drivers for a decrease in healthy ageing index scores would further support the application of the index and interpretation of scores. The index contains a greater proportion of biomedical and physical function items which may explain why there is an association between a decrease in healthy ageing index scores and pain. However in supplementary analysis (Table 5), a significantly greater number of deficits occurred with the onset of widespread pain in the psychological and social domains in addition to the biomedical and physical domains, indicating that the decrease in healthy ageing index score is down to the impact of pain across the domains of healthy ageing. It is possible that how healthy ageing is operationalized may lead to different findings [36], [37], however similar methods used in other ageing constructs (i.e. frailty) suggest that the composition of indices has little effect [38]. Although there is no gold standard for measuring healthy ageing, the constituents of the measures of healthy ageing in other studies (e.g. [39]) are the same constructs that are included in the measure used in this study. The sample for analysis included persons who responded at all three time points at 0, 3 and 6 years. Participants with complete data represented 30.7% of the baseline population of 9611. Whilst this may potentially affect the prevalence of pain and the distribution of healthy ageing, it is unlikely to have affected the estimates of the associations between the two; the association between widespread pain and healthy ageing index scores were similar in those included in the analysis (n = 2949) and in those who dropped out (n = 5826). In addition, sensitivity analysis was performed to investigate the impact of subject attrition and missing data, using probability-weighted analyses for survey data [27]. There were no differences in the observed associations between the original and weighted analyses. The generalisability of the study may be limited by the characteristics of the study population: the area covered by the study was more deprived on health, education, and employment, but with fewer barriers to housing and services, than England as a whole. The three-year gap between time points may miss some of the changes in status. Finally, there may be other confounders (e.g. current financial status) which may be important but which were not included in this study.

**Table 5:** Proportion of those who are free and experience the onset of widespread pain at 3 years who have an increase in the number of deficits in each of the 5 healthy ageing domains.

Domain	Free of widespread pain(n = 1622)	Onset of widespread pain(n = 365)	% difference (95% CI)	P-value
**Biomedical**	48.7%	60.0%	11.2 (5.6, 17.0)	<0.001
**Lay**	33.7%	36.4%	2.8 (−2.7, 8.2)	0.31
**Psychological**	18.1%	25.8%	7.6 (2.8, 12.5)	<0.001
**Physical function**	41.3%	49.9%	8.6 (2.9, 14.2)	0.003
**Social**	13.0%	20.0%	7.1 (2.6, 11.4)	<0.001

CI Confidence interval. Chi square test provides p values.

These findings provide evidence suggesting that the onset of widespread pain was associated with a decrease in healthy ageing in older adults. Prevention and treatment of pain is complex, particularly in older adults due to comorbidity and polypharmacy. This study suggests that further prospective cohort and intervention studies would be justified to establish whether early identification, improved treatment and prevention of pain prior to old age could promote healthy ageing, as well as to understand the mechanisms by which the onset of widespread pain increased the risk of unhealthy ageing.
